# Non-linear association between surgical duration and length of hospital stay in primary unilateral total knee arthroplasty: a secondary analysis based on a retrospective cohort study in Singapore

**DOI:** 10.1186/s13018-025-06267-0

**Published:** 2025-10-08

**Authors:** Jianxiang Zhu, Zengbing Xia, Jikang Min, Wenlin Hu, Heng Li, Chao Mei

**Affiliations:** 1 Department of Orthopedics, The First People’s Hospital of Huzhou, Huzhou, 313000 Zhejiang Province China; 2 Department of Endocrinology, The First People’s Hospital of Huzhou, Huzhou, 313000 Zhejiang Province China

**Keywords:** Total knee arthroplasty, Surgical duration, Length of stay, Non-linear relationship

## Abstract

**Background:**

The relationship between surgical duration and length of hospital stay (LOS) in total knee arthroplasty (TKA) remains incompletely understood. We investigated the potential associations and modulating factors influencing LOS.

**Methods:**

In this retrospective cohort study, we analyzed 2,394 patients undergoing primary unilateral total knee arthroplasty at Singapore General Hospital (2013–2014). Surgical duration served as the primary exposure, with LOS as the principal outcome. We employed multivariable linear regression models, including piecewise linear regression, to elucidate the relationship between surgical duration and LOS.

**Results:**

A significant non-linear association emerged between surgical duration and LOS. A critical inflection point was identified at 115 min, beyond which LOS increased substantially (adjusted *β* = 0.047, 95% CI: 0.018–0.076, *P* = 0.0015). Stratified analyses revealed nuanced effect modifications by anemia status and American Society of Anesthesiologists Physical Status (ASA-PS) scores. Patients with moderate-to-severe anemia and higher ASA-PS scores demonstrated markedly different response patterns, with more pronounced increases in hospitalization duration.

**Conclusion:**

Our findings demonstrate a complex, non-linear relationship between surgical duration and LOS in TKA. Anemia status and physiological reserve significantly modulate this association, suggesting the need for personalized perioperative management strategies. These insights may optimize surgical planning and resource allocation in orthopedic interventions.

**Supplementary Information:**

The online version contains supplementary material available at 10.1186/s13018-025-06267-0.

## Introduction

Total knee arthroplasty (TKA), recognized as the gold standard for treating end-stage knee osteoarthritis, has experienced a global annual increase in demand ranging from 5–10% [1, 2]. Despite significant advancements in surgical techniques and perioperative care, the length of hospital stay (LOS) continues to be a crucial factor influencing healthcare resource allocation and the efficiency of patient recovery. Extended LOS is closely linked with heightened risks of postoperative complications and increased healthcare costs, with estimates suggesting a 12% reduction in total expenses for each day of LOS saved [3–5]. Existing research has examined various factors affecting LOS, including patient demographics (such as age, BMI, and comorbidities), surgical techniques, and postoperative analgesia protocols [6–9].

While surgical duration represents a potentially modifiable perioperative variable, its precise relationship with LOS remains incompletely elucidated. Existing literature suggests that prolonged operative times may indirectly impact patient recovery through mechanisms including increased tissue trauma, systemic inflammatory responses, and extended anesthesia exposure [9, 10]. However, previous investigations have been constrained by methodological limitations, including sample heterogeneity and uncontrolled confounding factors such as surgical team expertise and intraoperative blood loss [11, 12]. Critically, a nuanced understanding of the independent association between surgical duration and hospital LOS could provide pivotal insights for optimizing operating room efficiency and developing personalized perioperative management strategies [13, 14]. Consequently, we performed a secondary analysis based on previously published data [15], to explore the potential association between surgical duration and LOS.

## Materials and methods

### Data sources

This retrospective population-based study employed data sourced from the Dryad Digital Repository (https://datadryad.org/stash/dataset/doi:10.5061/dryad.73250), a platform that offers unrestricted access to raw research datasets. The original study authors have transferred data ownership rights to the Dryad repository. In compliance with Dryad’s Terms of Service, we performed a secondary analysis on the data package under a novel hypothesis, ensuring adherence to the intellectual property rights of the original authors.

### Study population

This retrospective cohort study was conducted at Singapore General Hospital from January 2013 to June 2014 [15]. Initially, 2,622 patients underwent total knee arthroplasty (TKA) during this period. Following the application of exclusion criteria, 206 patients with bilateral TKA and 22 patients who required revision TKA were systematically excluded, resulting in a final study cohort of 2,394 participants who underwent primary unilateral TKA.

The study protocol received approval from the SingHealth Institutional Review Board (IRB), and the requirement for individual patient informed consent was waived due to the retrospective design of the study and the minimal risk posed to patient privacy. All data were anonymized and managed in strict accordance with institutional ethical guidelines.

### Variables

In the initial observational cohort, clinical records of patients undergoing total knee arthroplasty (TKA) were retrospectively retrieved from the institutional clinical information system, and subsequently archived in the data repository and analytics system. We obtained the variables from the Dryad Digital Repository. Surgical duration was designated as the independent variable, with LOS serving as the dependent variable. This study identified covariates as potential confounding factors affecting the relationship between surgical duration and LOS, drawing from existing literature. The comprehensive covariate analysis encompassed patient demographics (gender, age, race, body mass index), preoperative clinical characteristics, and operative details. Preoperative clinical assessment included smoking status, hemoglobin levels (evaluated using WHO’s gender-specific anemia criteria [16]), and American Society of Anesthesiologists Physical Status (ASA-PS) score [17]. The Revised Cardiac Risk Index (RCRI) [18] components were meticulously examined, including history of cerebrovascular accidents (CVAs), congestive cardiac failure (CCF), ischemic heart disease (IHD), diabetes mellitus (DM) on insulin, and preoperative creatinine levels exceeding 2 mg/dL. Operative variables comprised surgical duration, anesthesia type, day of surgical intervention [19], and perioperative blood transfusion. The latter was defined as transfusions occurring within a 4-week window (2 weeks before and after surgery).

LOS was operationalized as the temporal interval between hospital admission and discharge to the patient’s primary residence. Patients were predominantly admitted on the day of TKA, with rare exceptions of pre-operative admission for medical or social indications. Standard protocol involved discontinuing antiplatelet medications (except aspirin) for the recommended pre-surgical period. Notably, the use of intraoperative and postoperative interventions such as tranexamic acid infiltration, intravenous administration, joint drainage, and cell salvage were not uniformly standardized. Postoperative management followed a standardized hospital TKA protocol. This included thromboembolism chemoprophylaxis with 40 mg once-daily subcutaneous low molecular weight heparin (Clexane, Sanofi, Paris, France) initiated on the first postoperative day and discontinued at discharge. Routine physiotherapy commenced on the first postoperative day, regardless of weekend scheduling. Patient discharge was determined through a collaborative assessment by the surgeon and physiotherapist, confirming medical stability and functional capacity for home-based recovery. Discharge criteria included the patient’s ability to: (a) ascend several steps, (b) ambulate using a walking frame, and (c) achieve approximately 90° knee flexion of the operated joint. All these measures can minimize the influence of other factors on length of stay (LOS).

### Statistical analysis

The distribution of continuous variables was described with the use of the mean and the standard deviation (SD) or the median and the interquartile range [IQR], and frequencies and percentages are presented for categorical variables. We used χ2 (categorical variables), One-Way ANOVA test (normal distribution), or Kruskal-Whallis H test (skewed distribution) to test for differences among different operation duration (quartile). We used univariate and multivariate linear regression model to test the connection between surgical duration and LOS with three distinct models. Model 1 is the non-adjusted model with no covariates adjusted. Model 2 is the minimally-adjusted model with only sociodemographic variables adjusted (age, gender and race). Model 3 is the fully-adjusted model with covariates presented in Table 1 adjusted (age, gender, race, BMI, Hb, DM, creatinine > 2 mg/dL, OSA, IHD, CCF, CVA, ASA-PS score, perioperative blood transfusion, type of anaesthesia and the day of week the surgery was done). Because linear regression model based methods are often suspected for their inability to deal with non-linear models, nonlinearity between surgical duration and LOS were addressed using a Generalized Additive Model (GAM) and the smooth curve fitting (penalized spline method). If nonlinearity was detected, we firstly calculated the inflection point using recursive algorithm, and then constructed a two-piecewise linear regression model on both sides of the inflection point. The subgroup analyses were performed using stratified linear regression model. For continuous variable, we first converted it to a categorical variable according to the clinical cut point or tertlie, and then performed an interaction test. Tests for effect modification for those of subgroup indicators were followed by the likelihood ration test.

**Table 1:** Multivariable regression analysis of surgical duration and LOS

Exposure	Non-adjusted model β (95%CI), *P* value	Adjust I model β (95%CI), *P* value	Adjust II model β (95%CI), *P* value
Surgical duration	0.022 (0.014, 0.031) < 0.00001	0.022 (0.014, 0.031) < 0.00001	0.015 (0.007, 0.023) 0.00033
Surgical duration quartiles			
Q1	Reference	Reference	Reference
Q2	0.133 (-0.469, 0.735) 0.66486	0.227 (-0.369, 0.822) 0.45598	0.199 (-0.363, 0.761) 0.48841
Q3	0.538 (0.004, 1.072) 0.04844	0.487 (-0.042, 1.015) 0.07126	0.460 (-0.041, 0.961) 0.07205
Q4	0.973 (0.421, 1.525) 0.00056	1.036 (0.489, 1.583) 0.00021	0.644 (0.117, 1.170) 0.01663
*P* for trend	0.00020	0.00014	0.01026

***Abbreviations***: LOS, length of stay; CI, confidence interval

**Non-adjusted model**: No covariates were adjusted

**Adjust I model**: Age, gender and race were adjusted

**Adjust II model**: Age, gender, race, BMI, Hb, DM, creatinine > 2 mg/dL, OSA, IHD, CCF, CVA, ASA-PS score, perioperative blood transfusion, type of anaesthesia and the day of week the surgery was done were adjusted

To text the robustness of our results, we performed a sensitivity analysis. We converted surgical duration into a categorical variable according to the quartile, and calculated the *P* for trend in order to verify the results of operation duration as the continuous variable, and to examine the possibility of nonlinearity.

All the analyses were performed with the statistical software packages R (http://www.R-project.org, The R Foundation) and EmpowerStats (http://www.empowerstats.com, X&Y Solutions, Inc, Boston, MA). *P* values less than 0.05 (two-sided) were considered statistically significant.

## Results

### Baseline characteristics of selected participants

A total of 2,394 patients were enrolled and stratified into four quartiles (Q1-Q4) based on surgical duration, as presented in Table 2. The average patient age was 66.49 years, with 76.06% being female. Significant differences in baseline characteristics were noted across surgical duration groups. Age distribution was consistent (*P* = 0.007), but gender varied significantly (*P* = 0.019). Most patients were Chinese (84.08%), with stable ethnic proportions. The mean BMI was 27.74 kg/m², and hemoglobin levels averaged 13.08 g/dL. Diabetes prevalence increased with longer surgeries (Q1: 14.39% vs. Q4: 26.78%, *P* < 0.001), while other comorbidities like OSA, IHD, and CVA remained low. Regional anesthesia was used in 65.36% of cases, and general anesthesia in 33.29%. About 87.38% of patients were ASA-PS (2), indicating stable preoperative status. Significant differences were found in operative day distribution (*p* < 0.001), with higher blood transfusion rates in the Q4 group (7.12% vs. 3.73–4.80%, *P* = 0.033).

**Table 2:** Baseline characteristics of patients stratified by surgical duration quartiles

Surgical Duration	Q1 (30.00–60.00 min)	Q2 (65.00–70.00 min)	Q3 (75.00–90.00 min)	Q4 (95.00–225.00 min)	*P*-value
N	542	456	750	646	
**Demographics**					
Age, mean (SD), years	66.74 (8.29)	65.74 (8.42)	67.29 (8.12)	66.16 (8.10)	0.007^*^
Gender, n (%)					0.019^*^
Female	426 (78.60%)	355 (77.85%)	572 (76.27%)	462 (71.52%)	
Male	116 (21.40%)	101 (22.15%)	178 (23.73%)	184 (28.48%)	
Race, n (%)					0.475
Chinese	460 (84.87%)	382 (83.77%)	634 (84.53%)	537 (83.13%)	
Malay	37 (6.83%)	38 (8.33%)	43 (5.73%)	53 (8.20%)	
Others	45 (8.30%)	36 (7.89%)	73 (9.73%)	56 (8.67%)	
**Clinical Parameters**					
BMI, mean (SD), kg/m²	28.01 (4.81)	27.60 (4.99)	27.90 (4.71)	27.44 (4.46)	0.143
Hb, mean (SD), g/dL	13.07 (1.42)	13.04 (1.37)	13.10 (1.45)	13.09 (1.53)	0.901
Creatinine > 2 mg/dl, n (%)					0.312
No	490 (90.41%)	394 (86.40%)	655 (87.33%)	576 (89.16%)	
Yes	2 (0.37%)	6 (1.32%)	5 (0.67%)	5 (0.77%)	
**Lifestyle Factors**,** n (%)**					
Smoking					0.200
No	499 (92.07%)	415 (91.01%)	681 (90.80%)	572 (88.54%)	
Yes	43 (7.93%)	41 (8.99%)	69 (9.20%)	74 (11.46%)	
**Comorbidities**,** n (%)**					
DM					< 0.001^*^
No	464 (85.61%)	401 (87.94%)	606 (80.80%)	473 (73.22%)	
Yes	78 (14.39%)	55 (12.06%)	144 (19.20%)	173 (26.78%)	
DM on insulin					0.941
No	399 (73.62%)	337 (73.90%)	557 (74.27%)	490 (75.85%)	
Yes	8 (1.48%)	7 (1.54%)	15 (2.00%)	11 (1.70%)	
OSA					0.610
No	496 (91.51%)	420 (92.11%)	680 (90.67%)	581 (89.94%)	
Yes	46 (8.49%)	36 (7.89%)	70 (9.33%)	65 (10.06%)	
IHD					0.216
No	521 (96.13%)	429 (94.08%)	702 (93.60%)	614 (95.05%)	
Yes	21 (3.87%)	27 (5.92%)	48 (6.40%)	32 (4.95%)	
CCF					0.611
No	540 (99.63%)	451 (98.90%)	744 (99.20%)	641 (99.23%)	
Yes	2 (0.37%)	5 (1.10%)	6 (0.80%)	5 (0.77%)	
CVA					0.181
No	532 (98.15%)	452 (99.12%)	730 (97.33%)	634 (98.14%)	
Yes	10 (1.85%)	4 (0.88%)	20 (2.67%)	12 (1.86%)	
**Operative Details**					
Type of anaesthesia, n (%)					0.295
RA	361 (66.61%)	306 (67.11%)	489 (65.20%)	404 (62.54%)	
GA	178 (32.84%)	147 (32.24%)	250 (33.33%)	231 (35.76%)	
Others	3 (0.55%)	3 (0.66%)	11 (1.47%)	11 (1.70%)	
ASA-PS score, n (%)					0.275
1	33 (6.09%)	27 (5.92%)	50 (6.67%)	53 (8.20%)	
2	484 (89.30%)	402 (88.16%)	649 (86.53%)	546 (84.52%)	
3	25 (4.61%)	27 (5.92%)	51 (6.80%)	47 (7.28%)	
Day of week of operation, n (%)					< 0.001^*^
Monday	73 (13.47%)	70 (15.35%)	142 (18.93%)	112 (17.34%)	
Tuesday	178 (32.84%)	115 (25.22%)	154 (20.53%)	96 (14.86%)	
Wednesday	57 (10.52%)	76 (16.67%)	121 (16.13%)	156 (24.15%)	
Thursday	108 (19.93%)	106 (23.25%)	175 (23.33%)	146 (22.60%)	
Friday	89 (16.42%)	55 (12.06%)	122 (16.27%)	121 (18.73%)	
Saturday	37 (6.83%)	34 (7.46%)	36 (4.80%)	15 (2.32%)	
Perioperative blood transfusion, n (%)					0.033^*^
No	516 (95.20%)	435 (95.39%)	722 (96.27%)	600 (92.88%)	
Yes	26 (4.80%)	21 (4.61%)	28 (3.73%)	46 (7.12%)	
Length of Stay, median (IQR), days	4.00 (3.00–5.00)	4.00 (3.00–5.00)	4.00 (3.00–6.00)	5.00 (4.00–6.00)	< 0.001^*^

***Abbreviations***: BMI, body mass index; Hb, hemoglobin; DM, diabetes mellitus; OSA, obstructive sleep apnea; IHD, schaemic heart disease; CCF, congestive cardiac failure; CVA, cerebrovascular accidents; GA, general anaesthesia; RA, regional anaesthesia; ASA-PS, American Society of Anesthesiologist Physical Status; IQR, interquartile range; SD: standard deviation

*P*-values calculated using ANOVA for continuous variables, χ² test for categorical variables, and ^*^Kruskal-Wallis test for non-normally distributed data

### Univariate analysis of factors influencing LOS

Table 3 presents the univariate analysis results. Patients over 65 had a significantly longer LOS (*β* = 1.07, 95% CI: 0.68–1.46, *P* < 0.0001). Anemia severity affected LOS, with mild anemia increasing it (*β* = 1.30, 95% CI: 0.78–1.82, *P* < 0.0001) and moderate to severe anemia causing a greater increase (*β* = 2.06, 95% CI: 1.29–2.83, *P* < 0.0001). Elevated creatinine levels (> 2 mg/dL) led to the most significant LOS increase (*β* = 6.53, 95% CI: 4.29–8.76, *P* < 0.0001). Other factors prolonging stay included diabetes mellitus (*β* = 0.94, 95% CI: 0.44–1.43, *P* = 0.0002), ischemic heart disease (*β* = 1.71, 95% CI: 0.85–2.57, *P* < 0.0001), and congestive cardiac failure (*β* = 5.89, 95% CI: 3.66–8.13, *P* < 0.0001). General anesthesia and the highest ASA-PS score were also linked to longer stays. Perioperative blood transfusion was a strong predictor of extended hospitalization (*β* = 7.72, 95% CI: 6.89–8.55, *P* < 0.0001). The day of surgery also affected LOS, with variations noted on Monday.

**Table 3:** Univariate analysis of factors influencing LOS

Covariate	Statistics, *n* (%)	β(95%CI)	*P* value
**Demographics**			
Age, years			
≤ 65	1083 (45.24%)	Reference	
> 65	1311 (54.76%)	1.07 (0.68, 1.46)	< 0.0001^*^
Gender			
Female	1815 (75.81%)	Reference	
Male	579 (24.19%)	-0.15 (-0.60, 0.31)	0.5270
Race			
Chinese	2013 (84.09%)	Reference	
Malay	171 (7.14%)	-0.47 (-1.23, 0.28)	0.2194
Others	210 (8.77%)	0.22 (-0.47, 0.91)	0.5346
**Clinical Parameters**			
BMI, kg/m²			
≤ 25	710 (30.38%)	Reference	
> 25, ≤ 30	995 (42.58%)	0.19 (-0.28, 0.66)	0.4275
> 30	632 (27.04%)	-0.34 (-0.87, 0.18)	0.1988
Anaemia			
None	1827 (76.32%)	Reference	
Mild	403 (16.83%)	1.30 (0.78, 1.82)	< 0.0001^*^
Moderate/severe	164 (6.85%)	2.06 (1.29, 2.83)	< 0.0001^*^
Creatinine > 2 mg/dl			
No	2115 (88.35%)	Reference	
Yes	18 (0.75%)	6.53 (4.29, 8.76)	< 0.0001^*^
**Lifestyle Factors**			
Smoking			
No	2167 (90.52%)	Reference	
Yes	227 (9.48%)	-0.48 (-1.15, 0.18)	0.1526
**Comorbidities**			
DM			
No	1944 (81.20%)	Reference	
Yes	450 (18.80%)	0.94 (0.44, 1.43)	0.0002^*^
DM on insulin			
No	1783 (74.48%)	Reference	
Yes	41 (1.71%)	0.34 (-1.17, 1.84)	0.6611
OSA			
No	2177 (90.94%)	Reference	
Yes	217 (9.06%)	-0.60 (-1.28, 0.07)	0.0813
IHD			
No	2266 (94.65%)	Reference	
Yes	128 (5.35%)	1.71 (0.85, 2.57)	< 0.0001^*^
CCF			
No	2376 (99.25%)	Reference	
Yes	18 (0.75%)	5.89 (3.66, 8.13)	< 0.0001^*^
CVA			
No	2348 (98.08%)	Reference	
Yes	46 (1.92%)	1.22 (-0.20, 2.63)	0.0917
**Operative Details**			
Type of Anaesthesia			
RA	1560 (65.16%)	Reference	
GA	806 (33.67%)	0.84 (0.43, 1.25)	< 0.0001^*^
Others	28 (1.17%)	0.38 (-1.43, 2.18)	0.6808
ASA-PS score			
1	163 (6.81%)	Reference	
2	2081 (86.93%)	0.40 (-0.36, 1.16)	0.3074
3	150 (6.27%)	3.86 (2.80, 4.92)	< 0.0001^*^
Day of week of operation			
Monday	397 (16.58%)	1.30 (0.67, 1.92)	< 0.0001^*^
Tuesday	543 (22.68%)	0.97 (0.40, 1.55)	0.0009^*^
Wednesday	410 (17.13%)	0.66 (0.04, 1.28)	0.0383^*^
Thursday	535 (22.35%)	Reference	
Friday	387 (16.17%)	1.55 (0.91, 2.18)	< 0.0001^*^
Saturday	122 (5.10%)	-0.01 (-0.96, 0.94)	0.9864
Perioperative blood transfusion			
No	2273 (94.95%)	Reference	
Yes	121 (5.05%)	7.72 (6.89, 8.55)	< 0.0001^*^
Surgical duration (mins)			
< 60	322 (13.45%)	Reference	
60–89	1264 (52.80%)	0.10 (-0.49, 0.69)	0.7458
≥ 90	808 (33.75%)	0.73 (0.10, 1.35)	0.0226^*^

***Abbreviations***: BMI, body mass index; DM, diabetes mellitus; OSA, obstructive sleep apnea; IHD, schaemic heart disease; CCF, congestive cardiac failure; CVA, cerebrovascular accidents; GA, general anaesthesia; RA, regional anaesthesia; ASA-PS, American Society of Anesthesiologist Physical Status; LOS, Length of Stay

### Multivariable regression analysis of surgical duration and LOS

We conducted a comprehensive multivariable regression analysis to investigate the relationship between surgical duration and LOS, as presented in Table 1. In the initial unadjusted analysis, each unit increase in surgical duration was significantly associated with an increase in LOS *(β* = 0.022, 95% CI: 0.014–0.031, *P* < 0.0001). Following sequential adjustments for potential confounders, the association remained statistically significant, albeit with a slightly reduced effect size (Adjusted Model II: *β* = 0.015, 95% CI: 0.007–0.023, *P* = 0.00033). To further clarify this relationship, we stratified surgical duration into quartiles, designating the first quartile (Q1) as the reference category. Quartile analysis indicated that, compared to the shortest surgical duration group (Q1), the longest surgical duration group (Q4) exhibited a significant increase in the outcome indicator (Adjusted Model II: *β* = 0.644, 95% CI: 0.117–1.170, *P* = 0.01663). Trend analysis further corroborated a dose-response relationship between surgical duration and the outcome indicator (*P* for trend = 0.01026).

### Nonlinear relationship between surgical duration and LOS

**Fig. 1:**
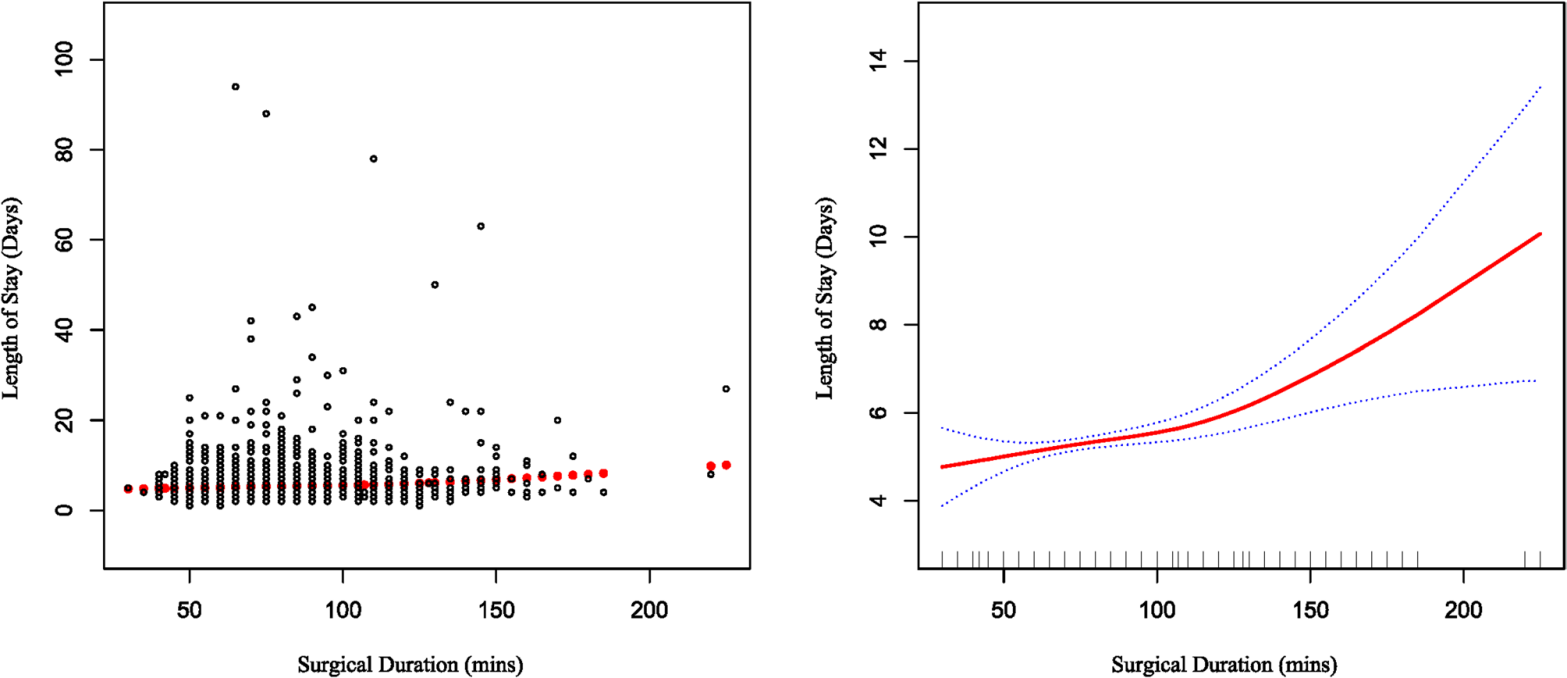
The association between surgical duration and LOS. (**A**) Each Black point represents a sample. (**B**) The solid red line represents the smooth curve fit between variables. Blue bands represent the 95% of confidence interval from the fit. Age, gender, race, BMI, Hb, DM, creatinine > 2 mg/dL, OSA, IHD, CCF, CVA, ASA-PS score, perioperative blood transfusion, type of anaesthesia and the day of week the surgery was done were adjusted

We utilized a piecewise linear regression model to examine the non-linear relationship between surgical duration and LOS, identifying a critical inflection point at 115 min, as presented in Table 4. After adjusting for potential confounding variables, a significant positive association was observed for observation times exceeding 115 min (adjusted *β* = 0.047, 95% CI: 0.018–0.076, *P* = 0.0015). The log-likelihood ratio test (*P* = 0.023) further corroborated the appropriateness of the piecewise linear model, indicating a significant non-linear dose-response relationship between surgical duration and LOS, as illustrated in Fig. 1.

**Table 4:** Association between surgical duration and LOS analyzed by GAM-based piecewise linear regression

Model	Non-adjusted β (95%CI)	*P*-value	Adjust model β (95%CI)	*P*-value
Inflection point (K)	115 min		115 min	
< K	0.012 (0.002, 0.022)	0.0243	0.008 (-0.002, 0.018)	0.1039
>K	0.069 (0.039, 0.099)	< 0.0001	0.047 (0.018, 0.076)	0.0015
LRT test	0.001		0.023	

***Abbreviations***: CI, confidence interval; LRT test, logarithmic likelihood ratio test; LOS, length of stay

**Non-adjusted model**: No covariates were adjusted

**Adjust model**: Age, gender, race, BMI, Hb, DM, creatinine > 2 mg/dL, OSA, IHD, CCF, CVA, ASA-PS score, perioperative blood transfusion, type of anaesthesia and the day of week the surgery was done were adjusted

### Subgroup analysis

**Fig. 2:**
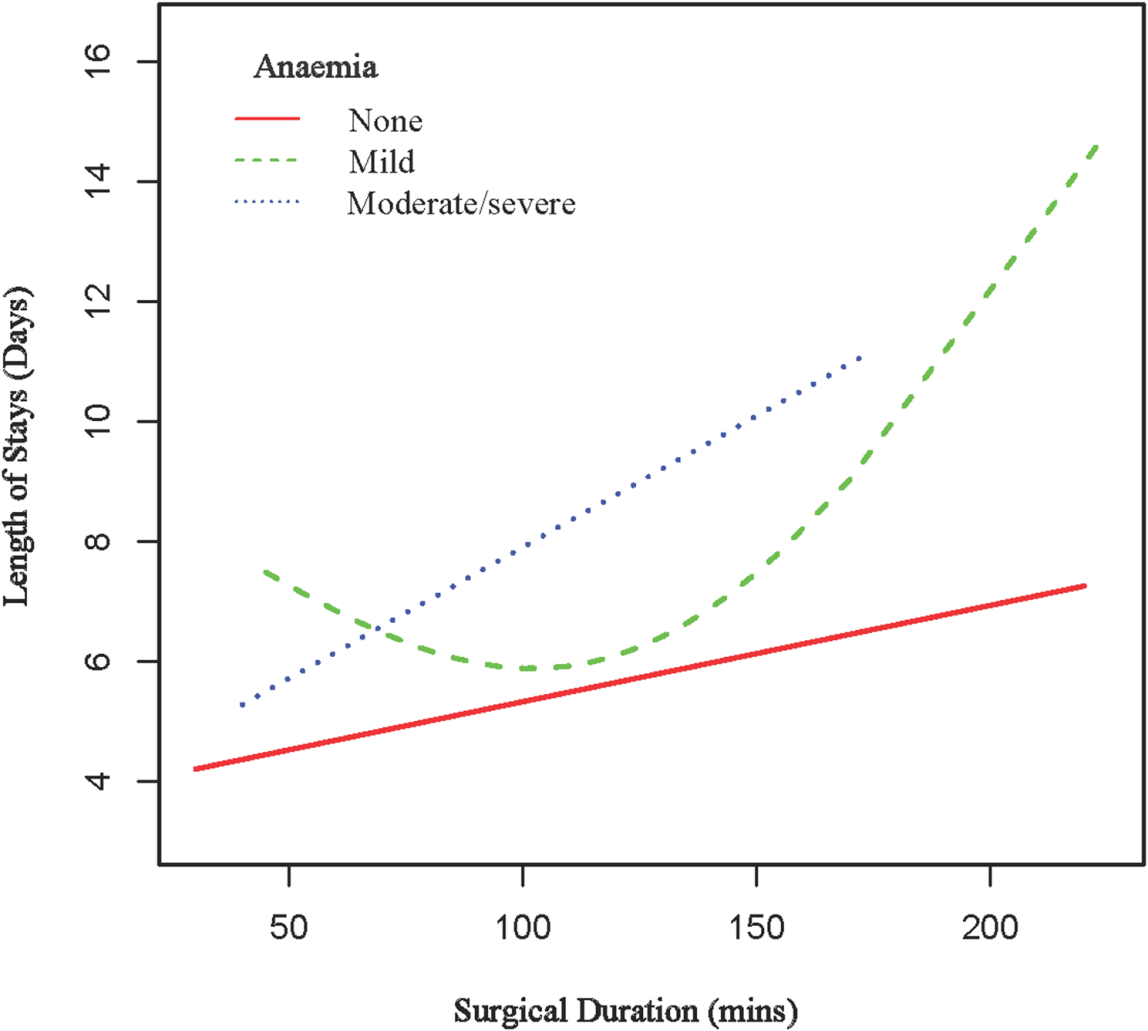
The association between surgical duration and LOS is stratified by anaemia. Age, gender, race, BMI, DM, creatinine > 2 mg/dL, OSA, IHD, CCF, CVA, ASA-PS score, perioperative blood transfusion, type of anaesthesia and the day of week the surgery was done were adjusted

**Fig. 3:**
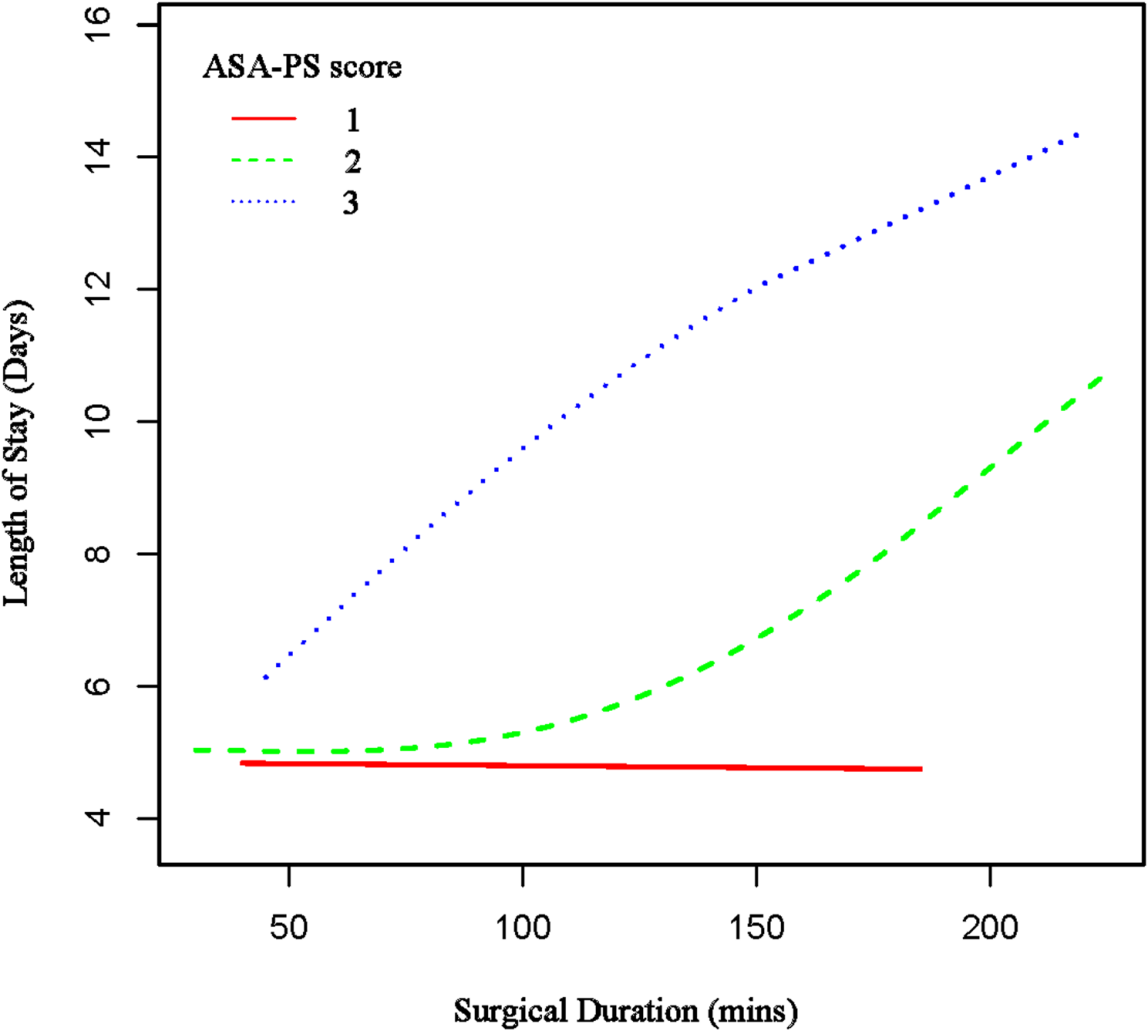
The association between surgical duration and LOS is stratified by ASA-PS score. Age, gender, race, BMI, Hb, DM, creatinine > 2 mg/dL, OSA, IHD, CCF, CVA, perioperative blood transfusion, type of anaesthesia and the day of week the surgery was done were adjusted

We performed a prespecified subgroup analysis to investigate potential effect modification by patient characteristics, as presented in Table 5. Within the cohort of 2,394 patients, surgical duration exhibited a consistent association with LOS across the majority of subgroups. Notably, two subgroups exhibited statistically significant interactions: anemia status (*P* for interaction = 0.0086) and ASA-PS score (*P* for interaction < 0.0001). Patients with moderate to severe anemia demonstrated a significantly different effect size (*β* = 0.050; 95% CI, 0.019–0.081) compared to those without anemia (*β* = 0.017; 95% CI, 0.008–0.026), as illustrated in Fig. 2. Similarly, the ASA-PS score revealed considerable variation in effect sizes, ranging from 0.001 for ASA-PS grade 1 to 0.111 for ASA-PS grade 3, as shown in Fig. 3. In contrast, other subgroups, including age, gender, race, body mass index, smoking status, diabetes, and various comorbidities, did not demonstrate statistically significant interactions (all *P* for interaction > 0.05).

**Table 5:** Effect size of surgical duration on LOS across prespecified and exploratory subgroups

Subgroup	*N*	β (95% CI)	*P* for interaction
**Demographics**			
Age			0.1879
≤65	1083	0.009 (-0.004, 0.021)	
>65	1311	0.019 (0.009, 0.030)	
Gender			0.4489
Female	1815	0.017 (0.008, 0.026)	
Male	579	0.010 (-0.007, 0.026)	
Race			0.2481
Chinese	2013	0.018 (0.009, 0.027)	
Malay	171	-0.004 (-0.035, 0.028)	
Others	210	-0.001 (-0.032, 0.031)	
**Clinical Parameters**			
BMI			0.7375
≤25	710	0.011 (-0.004, 0.025)	
>25, ≤ 30	995	0.017 (0.005, 0.029)	
>30	632	0.019 (0.002, 0.035)	
Anaemia			0.0086^*^
None	1827	0.017 (0.008, 0.026)	
Mild	403	-0.005 (-0.024, 0.014)	
Moderate/severe	164	0.050 (0.019, 0.081)	
Creatinine > 2 mg/dl			0.3947
No	2115	0.017 (0.009, 0.025)	
Yes	18	0.006 (-0.018, 0.031)	
**Lifestyle Factors**			
Smoking			0.7771
No	2167	0.015 (0.006, 0.023)	
Yes	227	0.019 (-0.009, 0.046)	
**Comorbidities**			
DM			0.5849
No	1944	0.014 (0.005, 0.023)	
Yes	450	0.020 (0.002, 0.037)	
DM on insulin			0.5572
No	1783	0.016 (0.006, 0.025)	
Yes	41	-0.033 (-0.129, 0.063)	
OSA			0.3378
No	2177	0.017 (0.008, 0.025)	
Yes	217	0.002 (-0.028, 0.031)	
IHD			0.5899
No	2266	0.015 (0.007, 0.023)	
Yes	128	0.026 (-0.014, 0.067)	
CCF			0.0798
No	2376	0.016 (0.008, 0.024)	
Yes	18	0.310 (-0.021, 0.642)	
CVA			0.3680
No	2348	0.015 (0.007, 0.024)	
Yes	46	-0.018 (-0.091, 0.055)	
**Operative Details**			
Type of Anaesthesia			0.9144
RA	1560	0.014 (0.004, 0.024)	
GA	806	0.015 (0.001, 0.029)	
Others	28	-0.006 (-0.105, 0.093)	
ASA-PS score			< 0.0001^*^
1	163	0.001 (-0.033, 0.035)	
2	2081	0.016 (0.007, 0.025)	
3	150	0.111 (0.079, 0.142)	
Perioperative blood transfusion			0.2453
No	2273	0.010 (0.002, 0.018)	
Yes	121	0.026 (-0.000, 0.053)	

***Abbreviations***: BMI, body mass index; DM, diabetes mellitus; OSA, obstructive sleep apnea; IHD, schaemic heart disease; CCF, congestive cardiac failure; CVA, cerebrovascular accidents; GA, general anaesthesia; RA, regional anaesthesia; ASA-PS, American Society of Anesthesiologist Physical Status; LOS, Length of Stay

## Discussion

In this extensive retrospective cohort study, encompassing 2,394 patients who underwent primary unilateral total knee arthroplasty, we conducted a systematic investigation into the relationship between surgical duration and LOS. The principal findings demonstrated that surgical duration was independently and significantly positively correlated with LOS. Additionally, we identified a threshold effect concerning surgical duration. Subgroup analyses further clarified the moderating effects of anemia status and ASA-PS score. To the best of our knowledge, this represents the first epidemiological study to provide definitive evidence of a nonlinear association between surgical duration and hospital stay in the context of primary unilateral TKA.

The impact of surgical duration on LOS in total knee arthroplasty remains incompletely understood. Fu et al.‘s [7] research on factors influencing LOS after total knee arthroplasty indicates that surgical duration is significantly correlated with LOS. In a comprehensive large-cohort investigation, Sodhi et al. [20] also demonstrated a significant correlation between surgical duration and LOS, with each 13-minute increment in operative time associated with an approximate one-day prolongation of LOS. However, in our analysis, we implemented a rigorous covariate adjustment strategy, encompassing a broader spectrum of potential confounding variables that may modulate LOS. We also employed a more sophisticated piecewise linear regression model, precisely locating the inflection point at 115 min, and uncovered more nuanced subgroup heterogeneity through interaction analyses of anemia status and ASA-PS score. Recently, robotic-assisted total knee arthroplasty (rTKA) challenges conventional understanding by reducing hospital length of stay despite extending operative duration by approximately 20 min [9, 21]. This counterintuitive outcome likely results from robotic technology’s precision in bone cutting and soft tissue preservation, which mitigates postoperative pain and accelerates patient recovery [22].

Evidence suggests that surgical interventions exceeding 120 min are associated with higher risks of postoperative complications, including deep vein thrombosis (DVT) and surgical site infections (SSI), which can significantly prolong LOS [10]. We speculate that prolonged surgical duration may indicate increased operative complexity, characterized by severe bone deficiency, challenging soft tissue balancing, or intraoperative complications, potentially resulting in enhanced tissue trauma and substantial blood loss, thereby prolonging LOS [23]. Additionally, extended procedures may exacerbate postoperative pain through prolonged soft tissue manipulation and tourniquet application, potentially impeding functional recovery [24].

As important modulators, anemia may delay hemoglobin recovery post-operatively, impairing wound healing and functional rehabilitation [25, 26]. The increased post-operative fatigue associated with anemia can lead to decreased adherence to early rehabilitation protocols, thereby prolonging the LOS [27]. Additionally, patients classified as ASA-PS grade 3 frequently have pre-existing functional limitations, such as chronic pain or cardiopulmonary dysfunction, which require extended recovery periods to achieve safe discharge criteria [23]. Careful preoperative management, encompassing hemoglobin optimization and holistic comorbidity assessment, may present a nuanced strategy for potentially reducing hospitalization duration in high-risk patients. However, further researches were needed to verify these speculations.

This study provides critical insights into perioperative management by revealing the non-linear relationship between surgical duration and hospital length of stay in total knee arthroplasty. We identified 115 min as a key inflection point, demonstrating that anemia status and ASA-PS score significantly modulate hospital stay, beyond simple linear associations reported in previous literature [20]. Our findings offer precise guidance for surgical scheduling, resource allocation, and patient risk stratification, particularly for high-risk patients. The results underscore the importance of precise surgical duration management and highlight the need for personalized perioperative strategies. Future research should investigate the mechanisms linking surgical duration to hospital stay across diverse surgical contexts and develop predictive models to optimize patient recovery and healthcare resource utilization.

This study offers novel insights through a robust methodological approach, analyzing 2,394 patients from Singapore General Hospital with rigorous selection criteria. Advanced statistical techniques identified a critical surgical duration inflection point at 115 min, utilizing progressive regression models with incremental confounder adjustment. As the first comprehensive investigation of moderating effects of anemia and ASA-PS scores on surgical duration and LOS, the research provides a pioneering framework for personalized perioperative risk stratification, systematically elucidating the complex relationship between surgical duration, patient characteristics, and hospitalization length.

This single-center retrospective cohort study has limitations. The sample of 2,394 patients, mostly Chinese (84.08%), may limit generalizability. While multi-level statistical adjustments were made, the observational design only shows associations between surgical duration and LOS, without establishing causality. Uncontrolled factors like surgical team skill [28], a multimodal analgesic regimen [29] and personalized rehabilitation may also affect outcomes. Excluding bilateral total knee arthroplasty and revision surgeries further restricts applicability. Future multi-center, prospective studies are needed to validate findings and improve population diversity.

## Conclusion

Our analysis demonstrates a significant non-linear correlation between surgical duration and length of hospital stay in primary unilateraltotal total knee arthroplasty, with a notable escalation in hospitalization length when operative time exceeds 115 min. Anemia and patient physiological status emerge as critical modulators of this relationship, offering potential pathways for personalized perioperative management.

## Electronic Supplementary Material

Below is the link to the electronic supplementary material.

Supplementary Material 1

## Data Availability

No datasets were generated or analysed during the current study.
