# Supplementary material for: Non-linear association between surgical duration and length of hospital stay in primary unilateral total knee arthroplasty: a secondary analysis based on a retrospective cohort study in Singapore
Source: J Orthop Surg Res. 2025 Oct 8;20:876. doi: 10.1186/s13018-025-06267-0 (PMC12505579; doi:10.1186/s13018-025-06267-0)
Supplement: Supplementary file 1 — Supplementary Material 1 [file 13018_2025_6267_MOESM1_ESM.docx]

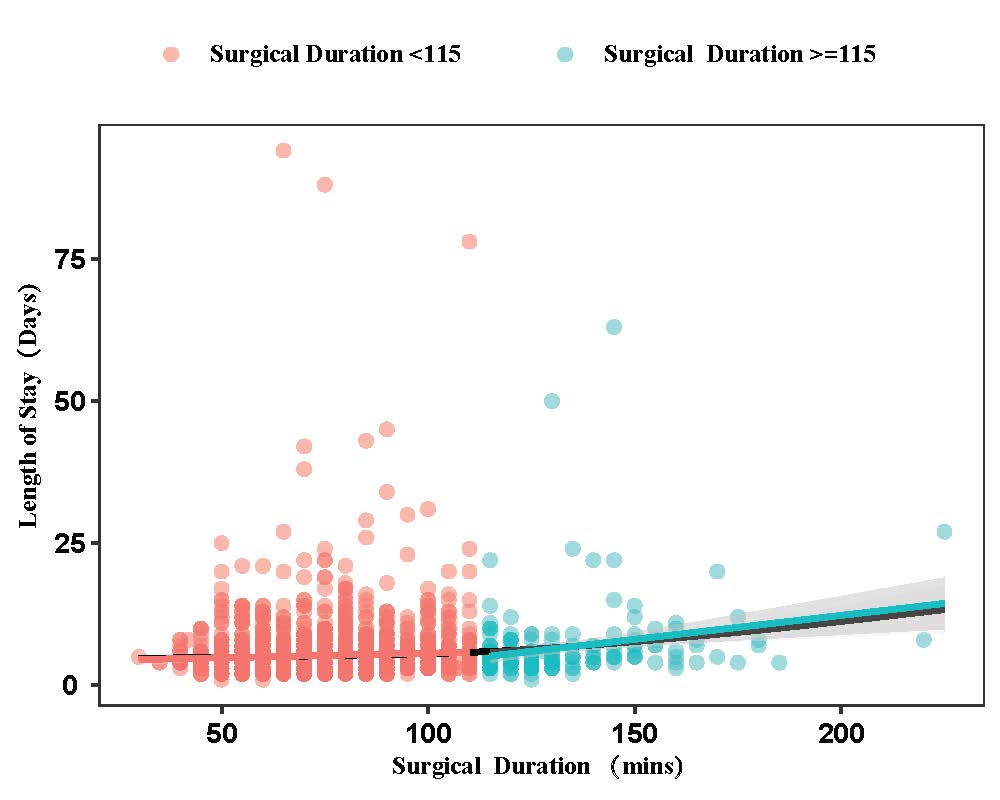


**Figure S1** Visualisation of the GAM-based piecewise linear regression for surgical duration versus length of stay
